# Corrections for Wavelength Variations in Precision Interferometric Displacement Measurements

**DOI:** 10.6028/jres.101.065

**Published:** 1996

**Authors:** Jack Stone, Steven D. Phillips, Gary A. Mandolfo

**Affiliations:** National Institute of Standards and Technology, Gaithersburg, MD 20899-0001; Hewlett Packard Co., Santa Clara, CA 95052-9952

**Keywords:** deadpath, interferometry

## Abstract

Precision interferometric displacement measurements require deadpath corrections to account for variations in wavelength during the course of the measurement. This paper discusses common errors in applying deadpath corrections and describes the correction necessary to fully account for variations in wavelength.

## 1. Introduction

Commercially available interferometer systems are used to measure the displacement of a moving reflector in terms of the wavelength (usually in air) of a laser or other light source. Corrections are required to account for wavelength variations during the measurement. (Wavelength variations most commonly arise from changes in the index of refraction of air due to changing atmospheric conditions—particularly pressure.) Wavelength variations are usually discussed in the context of “deadpath” corrections, which account for shifts in the interferometer zero position caused by wavelength changes. As discussed below, the standard deadpath correction is sensible only if displacement is computed using an appropriate value for the time-varying wavelength. Operating procedures for commercial interferometers may tend to result in an incorrect choice for the wavelength, so that error remain even after correcting for traditional deadpath errors. This paper attempts to clarify this issue by explicitly describing the deadpath correction procedure.

## 2. Analysis

Any interferometer for displacement measurements senses the phase difference between light reflected from a moving reflector and from a fixed reference reflector. A typical geometry for a single-pass interferometer is shown in Fig. 1. The phase difference changes by 2π for each half-wavelength displacement of the moving reflector; the net displacement can be determined by measuring the accumulated phase change as the mirror moves from its initial position to its final position.

When the distances from the beamsplitter to the moving reflector and reference reflector are *L* and *L*_R_ respectively, the phase difference between the light beams traversing the two arms of the interferometer is

$$\phi =\left(\frac{4\pi }{\lambda }\right)(L-L_{R})+(\phi_{M}-\phi_{R}).$$ (1)

Here *λ* is the wavelength of light in air, the quantity *ϕ*_M_ represents a phase shift of light in the measurement arm due to reflection and transmission through glass optics, and *ϕ*_R_ represents a similar quantity for the reference arm. We assume that the environment is sufficiently homogeneous that the wavelength is the same in any part of the air path.

Displacement is determined by measuring the change in the phase *ϕ*. Ignoring possible thermal drifts of the optics or thermal expansion of the reference arm, we may assume that *ϕ*_M_, *ϕ*_R_, and *L*_R_ are constant. The phase *ϕ* will then vary only in response to changes in *L* or *λ*:

$$d\phi =\left(\frac{4\pi }{\lambda }\right)dL-4\pi \left(\frac{L-L_{R}}{\lambda^{2}}\right)d\lambda .$$ (2)

A displacement interferometer effectively integrates the differential d*ϕ* and thus measures the total change in *ϕ* as the reflector moves from initial position *L*_i_ to a final position *L*_f_ (while the wavelength may also be changing from an initial value *λ*_i_ to a final value *λ*_f_). The resulting phase change is simply the difference between the initial and final values of *ϕ* [which could be obtained directly from Eq. (1)], independent of the path of integration; that is, the change in *ϕ* depends only on the initial and final values of *L* and *λ* and is independent of how these quantities vary at intermediate times.

$$\Delta \phi =4\pi \left\{\frac{L_{f}-L_{R}}{\lambda_{f}}-\frac{L_{i}-L_{R}}{\lambda_{i}}\right\}.$$ (3)

Equation (3) can be solved for the displacement Δ*L* = *L*_f_ − *L*_i_ in terms of the phase change Δ*ϕ* measured by the interferometer and the change in wavelength Δ*λ* = *λ*_f_−*λ*_i_:

$$\Delta L=\Delta \phi \left(\frac{\lambda_{f}}{4\pi }\right)+(L_{i}-L_{R})\frac{\Delta \lambda }{\lambda_{i}}.$$ (4)

This result may be interpreted as follows. The first term on the right converts a measured phase change to a displacement by multiplying the phase change Δ*ϕ* by *λ*_f_/4π, the displacement for each radian of phase. This term would clearly give a correct value for displacement if the wavelength had a constant value *λ*_f_ throughout the measurement. The second term can be thought of as a correction for motion of interference fringes past the initial position of the moving reflector, due to the change in wavelength. This term is the “deadpath” correction for the situation described here and depicted in Fig. 1; the quantity (*L*_i_ − *L*_R_) is the deadpath, and the deadpath correction accounts for an apparent shift in the position *L*_i_ caused by wavelength variations. (This will be referred to as a “zero-shift” error—an error arising from the apparent shift of the zero position *L*_i_—in the following discussion.) “Deadpath” is formally defined as the difference in optical path length between the measurement and reference arms at the beginning of the measurement, when the interferometer is set to zero [1].

Thus far we have explicitly considered only displacements of the moving reflector *away* from the beamsplitter, but Eq. (4) is also valid for a displacement *toward* the beamsplitter. In this case the second term on the right in Eq. (4) is again the deadpath correction with (*L*_i_ − *L*_R_) equal to the deadpath, but now the initial position *L*_i_ is the *farthest* distance of the moving reflector from the beamsplitter. Although this is in accord with the formal definition of deadpath, in practice “deadpath” is sometimes used to refer to the distance of closest approach of the moving reflector to the beam-splitter or, somewhat more precisely, to the distance of closest approach minus *L*_R_, where *L*_R_ is often negligibly small. The distinction between the formal definition of deadpath and this second usage of the term is not important for measurements with low accuracy (standard uncertainties, i.e., estimated standard deviations, larger than 1 or 2 × 10^−6^), but it is significant in high-accuracy applications.

With *either* meaning of deadpath an additional correction may be required to fully account for wavelength variations. The formal definition allows the two corrections to be clearly separated into two categories; the formal deadpath error can be interpreted unambiguously as a “zero-shift” error, independent of displacement, in contrast to errors that are proportional to displacement. In this paper we will adopt the formal definition of deadpath. In order to avoid possible confusion, we will refer to the deadpath error as a “zero-shift” error.

Equation (4) can be used to calculate displacement if the phase change Δ*ϕ* is known. If the interferometer readout gives displacement in units of *λ*/4 or some other fraction of a wavelength, the reading may easily be converted to a corresponding phase change Δ*ϕ*. Often the interferometer readout is given as a distance, in which case it is not quite so straightforward to determine the phase change. The distance displayed by commercial interferometers is calculated from Δ*ϕ* using

$$L_{0}=\Delta \phi \left(\frac{\lambda }{4\pi }\right).$$ (5)

Here *L*_0_ is the displacement shown by the interferometer display and *λ* is usually either *λ*_i_ or *λ*_f_. The value of *λ* depends on the nature of the interferometer and how it is used; this situation potentially creates confusion in calculating deadpath corrections. For example, some interferometers are equipped with external environmental sensors for determining the index of refraction; the value for the wavelength in air is continually updated as conditions change, and displacement is thus calculated with *λ = λ*_f_. Other interferometers compute *λ* from index of refraction information (velocity-of-light-compensation value) entered by the user before the displacement begins. If this information accurately reflects current atmospheric conditions, then the displacement is calculated with *λ* = *λ*_i_ in Eq. (5). Some users prefer to use an artificial value for the velocity-of-light compensation so that *λ* has some constant value independent of actual conditions. (For example, *λ* may be the vacuum wavelength.)

Substituting Eq. (5) into Eq. (4) yields a formula that corrects the readout *L*_0_ to give Δ*L*, the true displacement:

$$\Delta L=L_{0}+\left(\frac{\lambda_{f}-\lambda }{\lambda }\right)L_{0}+\left(L_{i}-L_{R}\right)\left(\frac{\lambda_{f}-\lambda_{i}}{\lambda_{i}}\right).$$ (6)

Here *λ* is the wavelength value used by the interferometer to calculate *L*_0_. As explained above, it is usually either the initial or final value of wavelength depending on the method of operation of the interferometer. If *λ* = *λ*_f_, only the rightmost term in Eq. (6) is required to correct the interferometer reading *L*_0_ for wavelength variations; this term is the zero-shift correction. However, it is clear from Eq. (6) that the zero-shift correction alone will not fully correct the interferometer reading when *λ* ≠ *λ*_f_. The second term on the right in Eq. (6) represents a correction proportional to the measured displacement that is required when the scale factor relating phase change to distance [*λ*/4p in Eq. (5)] is not calculated with *λ* = *λ*_f_.

In using either Eq. (4) or Eq. (6) to calculate displacement, several common errors must be avoided: (1) When calculating the zero-shift correction, the initial position *L*_i_ should not be confused with the distance of closest approach; (2) In order to be consistent with the conventions adopted in this paper, the interferometer should read positive for displacements of the moving reflector away from the beamsplitter and negative for displacements toward the beamsplitter; (3) Contrary to popular belief, using an average wavelength rather than the final wavelength *λ*_f_ in Eq. (4) does not improve accuracy; using an average wavelength will not give the correct displacement. Only the wavelengths at the beginning and end of the measurement are important. Values of wavelength at intermediate times have no effect on the final result. In fact, if *L*_i_ − *L*_R_ is zero, then displacement can be calculated from Eq. (4) knowing only a *single* wavelength *λ*_f_, or if *L*_f_ − *L*_R_ is zero only *λ*_i_ need be known. (The latter statement is not immediately obvious from Eq. (4).)

If the positive direction of the interferometer has been reversed, so that displacements of the moving reflector toward the beamsplitter are positive, then either the sign of the zero-shift correction must be changed or else the interferometer reading [Δ*ϕ* in Eq. (4) or *L*_0_ in the first two terms on the right in Eq. (6)] must be multiplied by −1.

## 3. Discussion and Conclusions

In summary, some care is required in making corrections for wavelength variations. The scale factor *λ*/4p required for calculating displacement from a phase change must be computed with *λ* = *λ*_f_, assuming that one calculates the zero-shift correction using the proper (formal) definition of deadpath. (In passing it may be noted that, if the zero-shift correction were to be computed using the distance of closest approach in place of the formally defined deadpath, then the appropriate scale factor would be given by the value of wavelength when the moving reflector is positioned farthest from the beamsplitter, independently of whether this is the initial or final position.) It is essential to use the appropriate scale factor or errors proportional to displacement will result. For example, if atmospheric pressure changes by 133 Pa (1 mm Hg) during a measurement, the computed displacement will be in error by 0.4 × 10^−6^Δ*L*. The resulting error is thus small but not negligible for the most demanding commercial and scientific applications of interferometry.

Two cases may be distinguished where corrections for wavelength variations are required:

1. Corrections are clearly needed for applications requiring relative errors smaller than 1 × 10^−6^. Although wavelength variations can often be eliminated by working in vacuum (so that the wavelength only varies due to imperfect laser stabilization), it is not always possible to do so. Special applications of interferometry require working under atmospheric conditions while keeping relative errors below a few parts in 10^8^ and it would be impossible to maintain this high level of accuracy without a proper treatment of wavelength variations.
2. Lower accuracy measurements may still require zero-shift corrections if the deadpath is very large relative to the displacement. For example, if a 1 mm displacement must be measured with a 1 m deadpath, a change in wavelength of 1 × 10^−6^*λ* will produce a zero-shift error that is 1 × 10^−3^ of the displacement. In this type of situation, with *L*_i_ approximately equal to *L*_f_, the common errors mentioned previously (confusion regarding the definition of deadpath or neglect of scale errors) are usually of negligible importance, but complete neglect of the zero-shift correction could be catastrophic.

Finally, it may be noted that the analysis given here is largely independent of interferometer details. Equation (4) or Eq. (6) can be used to correct the reading of any single-pass interferometer.

## Figures and Tables

**Fig. 1:**
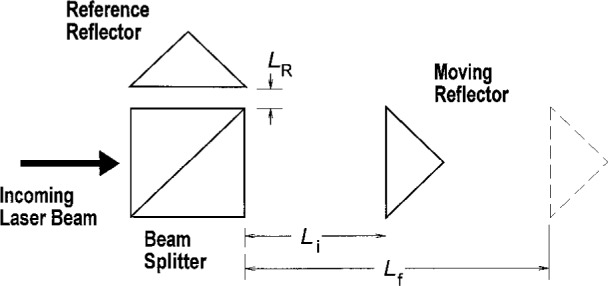
A typical Michelson interferometer with a cube beamsplitter and a moving corner cube to measure displacement. The length *L* of the measurement arm is *L* = *L*_i_ at the beginning of the displacement. The dashed triangle represents the position of the moving reflector at the end of the measurement (*L* = *L*_f_).
